# Computed tomography patterns of intracranial infarcts in a Ghanaian tertiary facility

**DOI:** 10.4314/gmj.v56i1.5

**Published:** 2022-03

**Authors:** Emmanuel K M Edzie, Klenam Dzefi-Tettey, Philip N Gorleku, Edmund K Brakohiapa, Peter Appiah-Thompson, Kwasi Agyen-Mensah, Michael K Amedi, Frank Quarshie, Evans Boadi, Joshua M Kpobi, Richard A Edzie, Abdul R Asemah

**Affiliations:** 1 Department of Medical Imaging, School of Medical Sciences, College of Health and Allied Sciences, University of Cape Coast, Cape Coast, Ghana; 2 Department of Radiology, Cape Coast Teaching Hospital, Cape Coast, Ghana; 3 Department of Radiology, Korle Bu Teaching Hospital, 1 Guggisberg Avenue, Accra, Ghana; 4 Department of Radiology, University of Ghana Medical School, Accra, Ghana; 5 Department of Surgery, ENT, School of Medical Sciences, College of Health and Allied Sciences, University of Cape Coast, Cape Coast, Ghana; 6 Department of Neurosurgery, School of Medical Sciences, College of Health and Allied Sciences, University of Cape Coast, Cape Coast, Ghana; 7 Department of Neurosurgery, Cape Coast Teaching Hospital, Cape Coast, Ghana; 8 Department of Radiology, 37 Military Hospital, Neghelli Barracks Liberation Road 37, Accra, Ghana; 9 African Institute for Mathematical Sciences (AIMS), Summerhill Estates, East Legon Hills, Santoe, Accra, Ghana

**Keywords:** Computed Tomography, Brain, Intracranial Infarcts, Patterns, Ghana

## Abstract

**Objective:**

To determine the Computed Tomography (CT) patterns of intracranial infarcts

**Design:**

A retrospective cross-sectional study.

**Setting:**

The CT scan unit of the Radiology Department, Cape Coast Teaching Hospital (CCTH), from February 2017 to February 2021

**Participants:**

One thousand, one hundred and twenty-five patients with non-contrast head CT scan diagnosis of ischaemic strokes, consecutively selected over the study period without any exclusions

**Main outcome measures:**

Patterns of non-contrast head CT scan of ischaemic strokes.

**Results:**

About 50.6% of the study participants were females with an average age of 62.59±13.91 years. Males were affected with ischaemic strokes earlier than females (*p<*0.001). The risk factors considered were, hyperlipidaemia (59.5%), hypertension (49.0%), Type 2 diabetes mellitus (DM-2) (39.6%) and smoking (3.0%). The three commonest ischaemic stroke CT scan features were wedge-shaped hypodensity extending to the edge of the brain (62.8%), sulcal flattening/effacement (57.6%) and loss of grey-white matter differentiation (51.0%), which were all significantly associated with hypertension. Small deep brain hypodensities, the rarest feature (2.2%), had no significant association with any of the risk factors considered in the study.

**Conclusion:**

Apart from the loss of grey-white matter differentiation, there was no significant association between the other CT scan features and sex. Generally, most of the risk factors and the CT scan features were significantly associated with increasing age.

**Funding:**

None declared.

## Introduction

Stroke is one of the many diseases worldwide that demand emergent attention. According to the World Stroke Organization (WSO), stroke has reached epidemic proportions and has also projected that globally, 1 out of 4 adults over 25 years will have a stroke in their lifetime.^1^

This tells the alarming nature of the stroke conditions and the need for more research to reduce its impact. Stroke was the largest cause of death globally after ischaemic heart diseases in 2016.^2^ The World Health Organization (WHO) has also reported that 15 million people suffer from strokes each year, out of which 5 million die and 5 million become permanently disabled.^3^ The overall age-standardised prevalence rate was 1300.6 per 100,000, and the incidence rate was 150.5 per 100,000 people in 2017.^4^ In the United States, it was reported that, on average, an individual in the United States suffers a stroke every 40 seconds.^5^

A stroke occurs when portions of the brain lose their blood and oxygen supply and eventually stop working.^1^,^6^ This can happen when blockage/narrowing occurs in the arteries (ischaemic) or rupture of a blood vessel in the brain (haemorrhagic), preventing the supply of blood and oxygen to the brain. Other types of strokes are transient ischaemic attack (TIA), which does not cause lasting symptoms as the two main types, and occurs when there is a temporary disruption of blood flow to the brain (usually resolves within 24 hours), and subarachnoid haemorrhage (SAH), which is caused by bleeding into the sub-arachnoid space.^7^ Ischaemic stroke is the most common type of stroke, making up 87% of all cases in the world.^8^

The introduction of imaging in medicine has contributed greatly to the success of healthcare delivery. This has made it possible to take brain images to detect areas where there is damage. There are many brain imaging techniques that are being used worldwide. These techniques include computed tomography (CT), functional magnetic resonance imaging, near-infrared spectroscopy, magnetoencephalogram, positron emission tomography, magnetic resonance imaging, and electroencephalography.^9^ A recent study in Ghana reported CT scan as one of the top four imaging modalities readily available and utilised in radiological practice.^10^ The CT scan of the brain produces an image of the brain due to the differential absorption of X-rays. It can be done with or without a contrast medium.^11^

Non-contrast enhanced computed tomography (NCCT) scan of the brain helps detect brain injury or acute trauma and bleeding resulting from a leaking or ruptured aneurysm in a patient with a severe headache of sudden onset. It can also identify a blood clot or brain parenchymal haemorrhage immediately after an acute stroke.^12^ NCCT generally helps diagnose ischaemic strokes much later, usually between 6–8 hours. After ruling out intracerebral bleeds, it is imperative to make an early diagnosis of ischaemic stroke so the appropriate life-saving interventions can be done, especially if the patient reports within the therapeutic window period (3–4.5 hours).^13^,^14^ Contemporary management of very early acute stroke has evolved as a result of the advances in neuro-imaging (advanced CT techniques like CT perfusion and angiography and MRI sequences) and treatments based on the “*time is brain*” concept. This has become more crucial since the advent of thrombolysis treatment, using endovascular therapy and tissue plasminogen activator.^15^ Conventional MRI sequences such as T2 weighted images carry little advantage over NCCT in sensitivity to stroke within the first hours. However, newer sequences, especially diffusion weighted MRI (DWI) and dynamic contrast bolus tracking perfusion MRI, also known as “perfusion weighted imaging” (PWI) offer considerable increases in diagnostic sensitivity and in contemporary acute stroke imaging, are better validated than CT techniques in defining pathophysiological parameters such as tissue viability in acute ischaemic stroke.^16^

The appearance of ischaemic stroke on CT is determined by the time interval between the occurrence of stroke and when the CT was done, hence the appearance varies from acute (<24 hours), to subacute (1–5 days), and chronic phases (weeks).^17^ This importance and many others have helped to emphasise the great contribution of CT scans in medicine. The risk factors for ischaemic strokes are reflective of those for atherosclerosis, and they include age, sex, stress, family history, oral contraceptives, smoking, alcohol abuse, diabetes mellitus, hypercholesterolaemia, and hypertension.^18^

The global prevalence of stroke in 2017 was 104.2 million people. Among the number of stroke victims, 82.4 million people suffered an ischaemic stroke, 17.9 million people suffered an intracerebral haemorrhage and 9.3 million people suffered a subarachnoid haemorrhage.^19^, ^20^ The prevalence of stroke has been reported to be high in Eastern Europe, the Middle East, and East and Central Asia. Countries in East Asia, Central Asia and Eastern Europe had the highest prevalence rates of ischaemic stroke in 2017. East and Central Asian countries recorded the highest prevalence in intracerebral haemorrhage and Japan recorded the highest prevalence in subarachnoid haemorrhage. Even though countries in Europe and Asia had the highest prevalence of stroke, they comparatively had low mortality rates. In sharp contrast, Africa, which is known to have had a low prevalence rate, is part of the region with the highest mortality rates. Reports in 2017 show that North Africa, Eastern Europe and Central Asia are the regions that had high mortality rates in ischaemic stroke.^21^, ^22^, ^23^ A study by Owolabi et al. showed that the impact of stroke in Africa tends to be escalating as the age-standardised prevalence rate is 981 per 100,000, the yearly stroke incidence rate with age-standardisation is 316 in every 100,000 people, and the fatality rate is 84%.^24^, ^25^

In Ghana, studies have also shown that, the occurrence of stroke has reached an epidemic stage, which could be as a result of the poor awareness of the risk factors of stroke.^26^, ^27^

Stroke in Ghana is the most important cause of disability, and it ranks among the top three causes of deaths, having mortality rates ranging from 126 to 150 per 100,000 people, and placing Ghana in the second category of countries with high mortality rates.^28^, ^29^, ^30^ With this high mortality rate in Ghana from stroke, this study aimed at reviewing CT patterns of areas of ischaemic stroke, to obtain the specific objectives of;

- Determining the demographic correlates of the population with ischaemic strokes.
- Ascertaining the common patterns of ischaemic strokes on CT scan.
- Finding a possible relationship of risk factors on CT scan patterns/features of ischaemic stroke.

## Methods

### Study design and Site

This was a retrospective study of all head CT scans of patients with ischaemic strokes, examined at the CT scan department, Cape Coast Teaching Hospital (CCTH), from February 2017 to February 2021. The CCTH serves as the major healthcare centre for specialised care in the Central Region of Ghana. It is a 400-bed capacity healthcare institution and currently the clinical training centre for the school of medical sciences, University of Cape Coast.

### Data collection

The ischaemic strokes diagnosed from all non-contrast head CT scans done during the study period were retrieved and evaluated from the Picture Archiving Communication System manufactured by IBM, Cambridge, MA, USA, by four imaging practitioners (1 radiographer and three radiologists) with a minimum of 12 years of experience in neuro-imaging. Radiological features considered for ischaemic strokes included the hyperdense middle cerebral artery (MCA) sign (focal hyperdensity of MCA on non-contrast brain CT), hypodensities with or without effacement of gyri and sulci, blurring of the internal capsules, loss of normal grey-white matter differentiation, in known vascular territory.^31^ Where there was disagreement in the findings, a consensus was attained by discussion. The brain CT scan images were obtained from the inferior-most part (base) of the skull to the vertex, with the following factors: tube current-exposure time 225mAs, tube voltage 120 kV, rotation time 0.75s, the slice thickness of 5mm and collimation 1x16, using a Toshiba Acquilion 16-slice multi-detector CT scanner, with model number, TSX-101A manufactured by Toshiba, Otawara, Japan. We reviewed the medical records and retrieved the available risk factors, including history of hyperlipidaemia, Type 2 diabetes mellitus (DM-2), hypertension, and smoking, from the Lightwave Health Management Information System for documentation.

In this study, hypertension was defined as sustained blood pressure (BP) elevation, BP > 140/90 mm Hg, according to the WHO and International Society of Hypertension guidelines.^32^ Type 2 diabetes mellitus (DM-2) was defined as a blood sugar level ≥ 200 mg/dL and hyperlipidaemia was defined as people with cholesterol lipoprotein ≥ 140 mg/dL, triglycerides > 200 mg/dL, high density lipoprotein < 40 mg/dL and/or total cholesterol > 200 mg/dL.^33^ Tobacco smoking was defined as any adult who has smoked 100 or more sticks of cigarettes in their lifetime.^34^ Some of the patients had more than one risk factor resulting in a total risk factor count of 1,700. Within the study period, 1,125 cases of ischaemic strokes were reviewed. The age and sex of patients with ischaemic strokes were also analysed. The ages of the participants were categorised as children (<18 years), active working age (“18–39 years” and “40–59 years”) and retired (≥60 years) based on the Ghana Statistical Service 2015 Labor Force report, and Ministry of Gender, Children and Social Protection in conjunction with UNICEF report.^35^, ^36^

### Statistical Analysis

The data obtained were organised for analysis with Statistical Package for Social Sciences (SPSS Inc. by IBM, Chicago, IL, USA) version 21.0 for Windows, to obtain the frequencies, tables, and to test for associations among the variables (CT scan features, age group, sex and the risk factors). A Chi-squared test of independence was used to check for associations among variables as indicated in the objectives and the level of significance was set at *p*≤0.05. We also examined the distribution of the ischaemic stroke CT scan features among the age groups and sex, as well as the proportions of risk factors of ischaemic stroke in our setting. The chart was obtained using LibreOffice Calc (version 1:6.1.5-3+deb10u6, developed by The Document Foundation). An independent sampled two-tailed students t-test was also used to test for the difference in means between male and female patients with ischaemic stroke after the assumption for normality check had been met.

### Ethical Considerations

Ethical clearance number (CCTHERC/EC/2020/099) for this study was obtained from the CCTH committee for ethical review. Informed consent was not required for this retrospective study. However, confidentiality and anonymity were ensured. This study conformed to the 1975 Declaration of Helsinki.

## Results

A total of 1,125 CT confirmed ischaemic stroke patients were included in the study, out of which 49.4% were males, and 50.6% were females.

The overall mean age of the study population was 62.59±13.91 years, with age range of 16–99 years. The majority (62.0%) of the patients were 60 years or more, followed by those who were aged 40–59 years (32.5%) and 18–39 years (5.3%). Only one patient (0.1%) was below the age of 18 years as shown in (Table 1). The mean age of the males in the study was 60.98±12.618 years and that of the females was 64.11±14.923 years (p<0.001).

**Table 1:** Demographic characteristics of patients

Variable	Count (%)
**Age;**	
Minimum Maximum Mean (SD)	16 99 62.59 (13.91)
**Age Group;**	
< 18 years 18–39 years 40–59 years ≥ 60 years	1(0.1%) 60(5.3%) 366(32.5%) 698(62.0%)
**Sex;**	
Male Female	556(49.4%) 569(50.6%)

**Figure 1:**
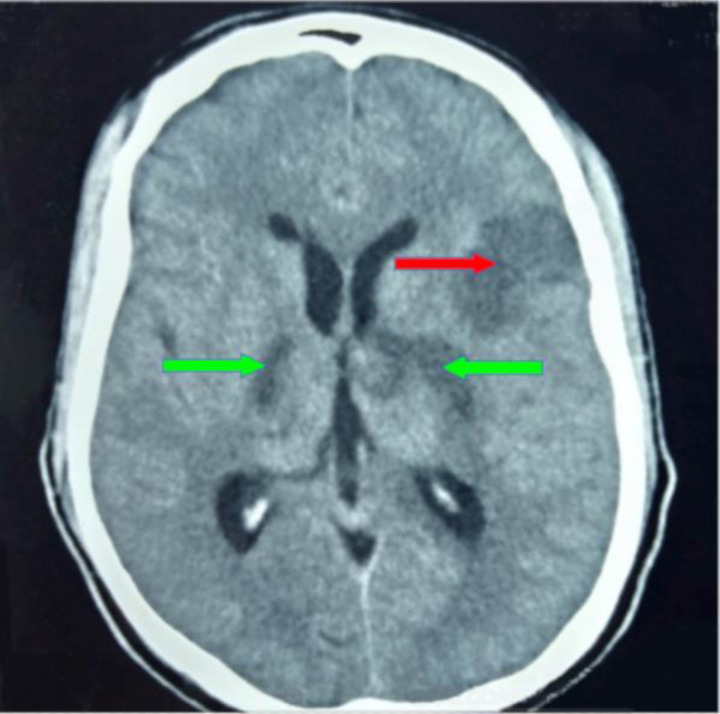
An axial non-enhanced CT scan of the brain showing an extensive area of hypodensity in the region of the left temporoparietal brain with associated sulcal flattening/effacement (wedge-shaped hypodensity extending to the edge of the brain) shown with red arrow, and at the basal ganglia areas (significant deep brain hypodensities) worse on the left shown with green arrows, in keeping with acute bilateral basal ganglia and left temporoparietal infarcts.

**Figure 2:**
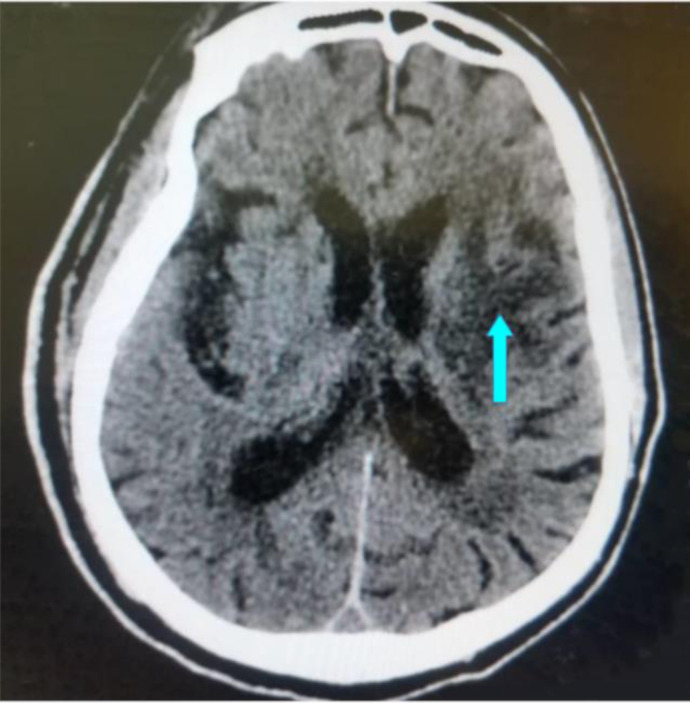
An axial non-enhanced CT scan of the brain showing an extensive area of hypodensity at the left basal ganglia (significant deep brain hypodensity with loss of grey-white matter differentiation and blurring of the internal capsule) shown with blue arrow in keeping with extensive acute right basal ganglia infarct.

**Figure 3:**
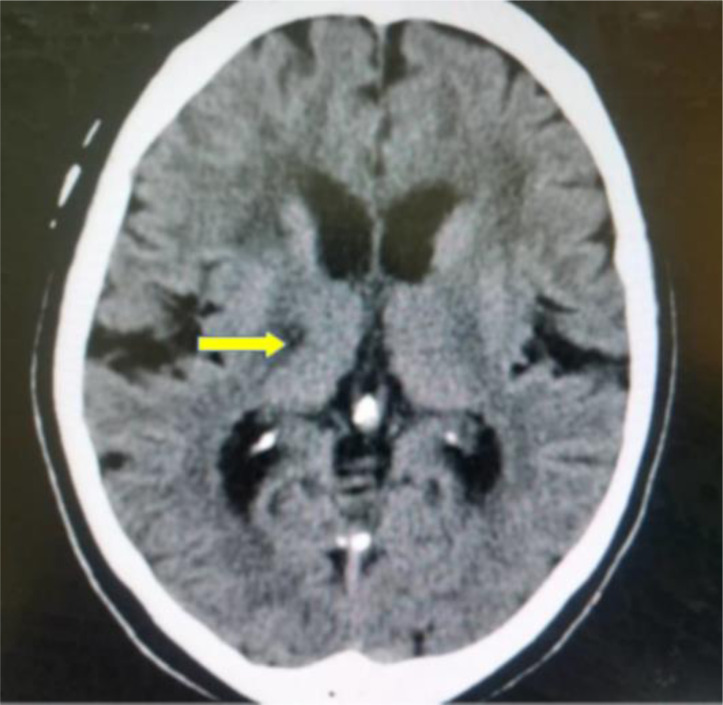
An axial non-enhanced CT scan of the brain showing an area of hypodensity at the right basal ganglia (small deep brain hypodensity) consistent with acute right basal ganglia infarct (shown with yellow arrow).

The majority of the patients with ischaemic stroke had hyperlipidaemia (59.5%), followed by hypertension (49.0%), DM-2 (39.6%) and only 3.0% had a smoking history. The most common ischaemic stroke CT scan features were wedge-shaped hypodensity extending to the edge of the brain (62.8%), followed by sulcal flattening/effacement (57.6%) (Figure 1) and loss of normal grey-white matter differentiation (51.0%) (Figure 2). The least recurring feature was small deep brain hypodensities (2.2%) (Figure 3). All these imaging features were more common among the aged (≥60 years). The distribution of CT scan features of ischaemic stroke among the age groups are shown in (Table 2). The significant majority of the patients who had loss of normal grey-white matter differentiation were females (53.7%, *p*=0.035), and 46.3% (*p*=0.035) were males. The distribution of the CT scan features of ischaemic stroke in relation to sex is also shown in (Table 2).

**Table 2:** Distribution of ischaemic stroke features with age and sex

Feature	Age Group				P-value	Total (%)
	< 18 years	18–39 years	40–59 years	≥ 60 years		
**Loss of Normal Grey-white Matter Differentiation**						574(51.0%)
Present	0(0.0%)	8(1.4%)	143(24.9%)	423(73.7%)	<0.001*	
Not Present	1(0.2%)	52(9.4%)	223(40.5%)	275(49.9%)		
**Blurring of the Internal Capsule**						146(13.0%)
Present	0(%)	6(4.1%)	31(21.2%)	109(74.7%)	0.007*	
Not Present	1(0.1%)	54(5.5%)	335(34.2%)	589 (60.2%)		
**Sulcal Flattening/Effacement**						648(57.6%)
Present	1(0.2%)	24(3.7%)	209(32.3%)	414(63.9%)	0.024*	
Not Present	0(0.0%)	36(7.5%)	157(32.9%)	284(59.5%)		
**Wedge-shaped Hypodensity extending to the edge of the brain**						706(62.8%)
Present	1(0.1%)	33(4.7%)	231(32.7%)	441(62.5%)	0.473	
Not Present	0(0.0%)	27(6.4%)	135(32.2%)	257(61.3%)		
**Significant Deep Brain Hypodensities**						425(37.8%)
Present	0(0.0%)	28(6.6%)	142(33.4%)	255(60.0%)	0.316	
Not Present	1(0.1%)	32(4.6%)	224(32.0%)	443(63.3%)		
**Small Deep Brain Hypodensities**						25(2.2%)
Present	0(0.0%)	0(0.0%)	3(12.0%)	22(88.0%)	0.023*	
Not Present	1(0.1%)	60(5.5%)	363(33.0%)	676 (61.5%)		

**Feature**	**Sex**				**P-value**	
	**Male**		**Female**			

**Loss of Normal Grey-white Matter Differentiation**						574(51.0%)
Present	266(46.3%)		308(53.7%)		0.035*	
Not Present	290(52.6%)		261(47.4%)			
**Blurring of the Internal Capsule**						146(13.0%)
Present	63(43.2%)		83(56.8%)		0.104	
Not Present	493(50.4%)		486(49.6%)			
**Sulcal Flattening/Effacement**						648(57.6%)
Present	318(49.1%)		330(50.9%)		0.785	
Not Present	238(49.9%)		239(50.1%)			
**Wedge-shaped Hypodensity extending to the edge of the brain**						706(62.8%)
Present	350(49.6%)		356(50.4%)		0.894	
Not Present	206(49.4%)		213(50.8%)			
**Significant Deep Brain Hypodensities**						425(37.8%)
Present	211(49.3%)		214(50.4%)		0.906	
Not Present	345(49.3%)		355(50.7%)			
**Small Deep Brain Hypodensities**						25(2.2%)
Present	9(36.0%)		16(64.0%)		0.175	
Not Present	547(49.7%)		553(50.3%)			

* Statistically significant

**Footnote**: There was no occurrence of hyperdense middle cerebral artery sign during our study period.

Most of the patients with hypertension, DM-2, and hyperlipidaemia were significantly 60 years or more, constituting 76.3% (*p*<0.001), 78.3% (*p*<0.001), and 59.1% (*p*<0.001), respectively. Smoking was found not to be significantly associated with the age groups (*p*=0.212). The ischaemic stroke risk factors and their associations with the age groups are shown in (Tabl**e** 3). Comparatively, more females had hypertension (53.3%, *p=*0.011), and hyperlipidaemia (53.4%, *p=*0.024) than males.

Most of the patients with a smoking history were males (97.1%, *p*<0.001). DM-2 was not significantly associated with sex (*p*=0.874).

Hyperlipidaemia was significantly associated with only one CT scan feature, thus losing normal grey-white matter differentiation (*p*=0.023). Only 29.4% of the patients who had a loss of normal grey-white matter differentiation (*p*=0.027) and 41.2% who had sulcal flattening/effacement (*p*=0.047) had a smoking history. The remaining results for the association test between the risk factors and the CT scan features are shown in (Table 3).

**Table 3:** Association of ischaemic stroke risk factors with age, sex and CT scan features

Variable	Hypertension		DM-2		Hyper		Smoking	
	Yes	P-value	Yes	P-value	Yes	P-value	Yes	P-value
**Age Group**								
< 18 years	0 (0.0%)	<0.001*	0 (0.0%)	<0.001*	0 (0.0%)	<0.001*	0 (0.0%)	0.212
18–39 years	5 (0.9%)		37 (7.9%)		13 (1.9%)		5 (14.7%)	
40–59 years	127 (22.8%)		64 (13.7%)		260 (39.0%)		11 (32.4%)	
≥ 60 years	425 (76.3%)		365 (78.3%)		394 (59.1%)		18 (52.9%)	
**Sex**								
Male	254 (45.6%)	0.011*	229 (49.1%)	0.874	311 (46.6%)	0.024*	33 (97.1%)	<0.001*
Female	303 (54.4%)		237 (50.9%)		356 (53.4%)		1 (2.9%)	
**Features**								
**Loss of Normal Grey-white Matter Differentiation**								
Present	321 (57.6%)	<0.001*	252 (54.1%)	0.085	359 (53.8%)	0.023*	11 (32.4%)	0.027*
Not Present	236 (42.4%)		214 (45.9%)		308 (46.2%)		23 (67.6%)	
**Blurring of the Internal Capsule**								
Present	79 (14.2%)	0.234	74 (15.9%)	0.015*	92 (13.8%)	0.326	4 (11.8%)	0.829
Not Present	478 (85.8%)		392 (84.1%)		575 (86.2%)		30 (88.2%)	
**Sulcal Flattening/Effacement**								
Present	340 (61.0%)	0.021*	256 (54.9%)	0.128	387 (58.0%)	0.730	14 (41.2%)	0.049*
Not Present	217 (39.0%)		210 (45.1%)		280 (42.0)		20 (58.8%)	
**Wedge-shaped Hypodensity extending to the edge of the brain**								
Present	368 (66.1%)	0.023*	270 (57.9%)	0.005*	403 (60.4%)	0.051	18 (52.9%)	0.229
Not Present	189 (33.9%)		196 (42.1%)		264 (39.6%)		16 (47.1%)	
**Significant Deep Brain Hypodensities**								
Present	191 (34.3%)	0.017*	204 (43.8%)	<0.001*	262 (39.3%)	0.210	16 (47.1%)	0.257
Not Present	366 (65.7%)		262 (56.2%)		405 (60.7%)		18 (52.9%)	
**Small Deep Brain Hypodensities**								
Present	17 (3.1%)	0.062	10 (2.1%)	0.884	14 (2.1%)	0.735	0 (0.0%)	0.213
Not Present	540 (96.9%)		456 (97.9%)		653 (97.9%)		34 (100%)	

* Statistically significant

## Discussion

Stroke has been reported to have affected more females than males because of the increase in longevity and the fact that stroke event rates increase in the oldest age groups, as shown in this study.^37^, ^38^ This could be the reason why the number of patients in our study increased substantially with increasing age and most of them being females (50.6%) as shown in (Table 1), even though studies have shown that, estrogens are extremely neuroprotective at several levels, including curtailment of stroke risk factor pathology through antiatherogenic effects in the vasculature and the control of adipogenesis in the discourse of ischaemic stroke.^39^, ^40^ This finding is similar to the findings of Wiredu et al., who reported the majority (51.89%) of the participants with ischaemic stroke being females,^41^ though the reports from other studies were contradictory to this finding.^38^, ^42^

A study by Yunusa et al., to determine the patterns of CT findings of the brain in stroke patients in Nigeria, found that the prevalence of stroke decreases with increasing age, especially after 40 years.^43^

Some studies have reported that hypertension is the most frequent risk factor seen among patients with ischaemic stroke.^18^,^33^ Their findings could be due to increased pain, psychological stress, urinary retention, elevated intracranial pressure and hypoxaemia.^44^ In our study, we found that more than half of the patients with cerebral infarcts had hyperlipidaemia constituting about 59.5%, followed by hypertension (49.0%), contrary to their findings. However, in a study by Boot et al., it was also reported that about 50–60% of patients with ischaemic stroke had hyperlipidaemia.^45^ This could be due to the increasing consumption of saturated carbohydrates, fats, and lack of exercise. It has been reported in Ghana that most people do not like exercising and would choose to use public transport than to walk to cover a relatively short distance.^46^ Studies have also shown that the use of pesticides also increases lipid levels, which could be a contributing factor since most Ghanaians engage in the agriculture business.^46^, ^47^, ^48^

Assessing the commonest radiological features of intracerebral infarcts among the age group, we found that the top three most common features were wedge-shaped hypodensity extending to the edge of the brain, sulcal flattening/ effacement (Figure 1) and loss of normal grey-white matter differentiation (Figure 2), which were all more prevalent among those 60 years or more. The radiological features of ischaemic strokes were more common in females than males (Table 2). For instance, loss of normal grey-white matter differentiation was more common in females (53.7%, *p*=0.035). Other studies have also reported these radiological features, who also referred to these ischaemic stroke features as the early CT findings of global central nervous system hypoperfusion.^49^, ^50^

Our study also showed that the majority of the patients with hypertension, Type-2 DM and hyperlipidaemia were females, which has also been corroborated by Yao et al.^51^ However, these findings are contrary to what was reported by Ojha et al.^52^ It is worthy of note that, both authors agreed to the fact that, smoking, as a risk factor of ischaemic stroke, is more common in males, as also reported in our study (Table 3). Most of the risk factors included in this study (hyperlipidaemia, hypertension, and DM-2) were significantly more common in those 60 years or older (*p<*0.001) (Table 3), similar to what was reported in India by Ojha et al.^53^

Our study also found that a majority of the patients who had loss of normal grey-white matter differentiation, which is usually due to the effect of cytotoxic oedema,^50^ were hypertensive (57.6%, *p*<0.001). This correlation was difficult to decipher, but other studies have reported the predictive effect of hypertension on the grey-white matter volume, contrary to our findings.^54^ Even though hyperlipidaemia was the commonest risk factor, it was not significantly associated with most of the CT scan features, it was only associated with loss of normal grey-white matter differentiation (*p*=0.023) (Table 3).

Studies have reported that females develop ischaemic strokes at an older age than males.^51^, ^55^ In a study by Appelros et al., it was reported that females get their strokes on an average of 4.3 years later than males. This finding is similar to our results. Our results showed that, males get an ischaemic stroke at a comparatively early age than females (*p*<0.001), with a significant average age difference of 3.13 years.

### Limitations

Other risk factors were not considered in this study and this could be an area for further research. Early or minor infarcts could have been missed since CT angiography and CT perfusion are unavailable in our setting. Again, the time interval from the onset of the stroke to the acquisition of the CT scan was also not included because of its inaccessibility from the LHMIS.

## Conclusion

The three commonest CT scan features of ischaemic stroke were wedge-shaped hypodensity extending to the edge of the brain, sulcal flattening/effacement and loss of normal grey-white matter differentiation, which were all significantly associated with hypertension. Apart from the loss of normal grey-white matter differentiation, there was no significant association between the other features and sex.
